# Computerized dynamic occlusal analysis and its correlation with static characters in post-orthodontic patients using the T-Scan system and the ABO objective grading system

**DOI:** 10.1186/s12903-023-02868-5

**Published:** 2023-05-22

**Authors:** Menglin Wang, Le Liu, Xihua Ma, Xiang Jin, Zhenbao Zhang, Xiangmin Jia, Jiadong Fan, Haoning Tang, Yanfeng Li

**Affiliations:** 1 Medical School of Chinese PLA, Beijing, People’s Republic of China; 2 Department of Stomatology, the Fourth Medical Centre, Chinese PLA General Hospital, No.51 Fucheng Road, 100048 Beijing, China

**Keywords:** Post-orthodontic, Static occlusion, Dynamic occlusion, Correlation, ABO-OGS, T-Scan

## Abstract

**Objectives:**

This study was conducted to detect the overall performance of both static and dynamic occlusion in post-orthodontic patients using quantified methods, and to ascertain the correlation between the two states of occlusion.

**Materials and methods:**

A total of 112 consecutive patients evaluated by ABO-OGS were included in this study. Based on the pre-treatment Angle’s classification of the malocclusion, samples were divided into four groups. After removing orthodontic appliances, each patients underwent the American Board of Orthodontic objective grading system (ABO-OGS) and T-Scan evaluations. All the scores were compared within these groups. Statistical evaluation included reliability tests, multivariate ANOVA, and correlation analyses (p < 0.05 was considered significant).

**Results:**

The mean ABO-OGS score was satisfactory and did not differ by Angle classifications. The indices making substantial contributions to ABO-OGS were occlusal contacts, occlusal relationships, overjet, and alignment. Disocclusion time in post-orthodontic patients was longer than normal. Occlusion time, disocclusion time, and force distribution during dynamic motions were considerably influenced by static ABO-OGS measurements, especially occlusal contacts, buccolingual inclination, and alignment.

**Conclusion:**

Post-orthodontic cases that passed the static evaluation of clinicians and ABO-OGS may nevertheless be left with dental casts interference in dynamic motions. Both static and dynamic occlusion should be extensively evaluated before ending orthodontic treatment. Further research is needed on dynamic occlusal guidelines and standards.

## Introduction

Orthodontic treatment aims to establish an ideal or normal tooth alignment, functional occlusion, aesthetic appearance, and stability. The function of the masticatory system is determined in large part by dental occlusion. And an ideal occlusion is comprised of both static occlusion and dynamic occlusion to help achieve treatment goals.

**Fig. 1:**
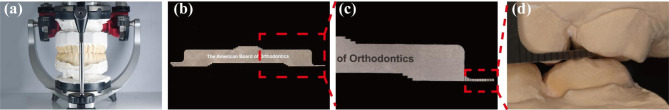
The analysis of static model on full adjustment articulator (PROTAR evo7, Kavo, German) (a). The uniform measuring gauge of the ABO-OGS system (b). This portion of the gauge was used to measure discrepancies in alignment, overjet, occlusal contacts, interproximal contacts, and occlusal relationships. It is in 1 mm increments and the width of the gauge is 0.5 mm (c). The measurement of occlusal contact on the labial side using the specific gauge (d). The ABO measurement tool used was purchased from the ABO official website: AmericanBoardOrtho.com. The manufacturer information of the measuring tape is: Bender Support, Bender.Inc., Fenton, USA

Static occlusion is characterized as the alignment of teeth and the static morphological relationship of the upper and lower dentition during inertial occlusion. Since the 1970s, several static standards have been proposed to evaluate the outcome of orthodontic [1], including Angle’s standard occlusion, six keys to normal occlusion by Andrews, the PAR index, the ICON [2, 3] and others. However, the result of the same case can differ dramatically depending on the examiner’s perspective and expertise. The American Board of Orthodontics introduced the Objective Grading System (ABO-OGS) in 1994 with an attempt to offer more neutral and reliable clinical [4]. While the severity of discordant was assessed and recorded by assigning different scores to eight categories of orthodontic cases, the reliability of different examinees was enhanced by using the same specific measuring gauge (Fig. 1b), and objective grading guidance. Seven categories including alignment, marginal ridges, buccolingual inclination, occlusal relationships, occlusal contacts, overjet, and interproximal contacts were measured and scored on dental casts of completed patients. Root angulation was assessed through radiographs. All of the indicators were examined and scored using objective, valid, and repeatable scoring procedures that eliminate subjective influence in scoring to the best extent [5]. ABO-OGS is a fundamental part of the American Board of Orthodontic clinical examination and is currently recognized as the standard index for orthodontic case [6].

Occlusal contact does not just refer to static occlusion, it also includes dynamic occlusion. The goal of dynamic occlusion evaluation is to see if and where early contact, atypical contacting points, and occlusion interference occur during mandibular dynamic motions. Articulating paper, foils, wax films, silicone rubber and study models are all common clinical procedures for examining aberrant occlusal [7]. These approaches, however, are unable to virtually recreate the complex dynamic motions of mandible. The composition of the materials utilized, such as articulating paper and wax, can be quickly altered by the oral environment, such as saliva and [8]. The operation of clinicians and the incorrect subjective interpretation of articulating paper marks left on teeth surfaces can cause false results in dynamic occlusion assessment. The dentists were only 12.8% of the time correction for picking high and low force contact areas by visual inspection of marks left on the teeth, according to Kerstein et [9]. Therefore, these procedures were insufficiently objective and repeatable.

**Fig. 2:**
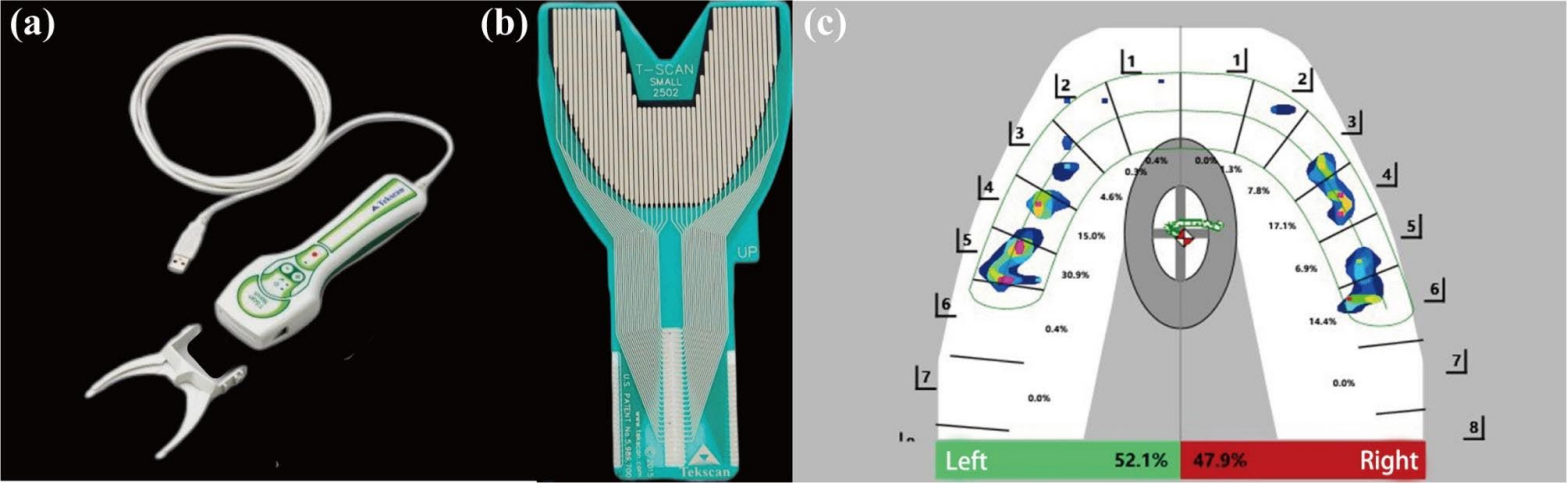
The T-Scan 10 recording hardware components include the Novus recording handle (a) and the high-definition specific sensor thin film (b). T-Scan recorded force and timing data of movements and could offer playback for further analysis and diagnosis. The distribution of force for each tooth is presented on the dental arch-shaped graphic (c)

The T-Scan technology has been developed since the 1980s and may provide a more accurate and objective assessment with the help of [10]. By computer analysis of information obtained from a flexible pressure-sensitive film (Fig. 2b), the T-Scan system was designed to investigate and record the time-varying changes of occlusal contacts during mandibular [11]. The T-Scan system was the first system to review the variation of occlusal contacts over time. On the computer, the period of tooth contact and separation can be recorded, evaluated, and presented intuitively. If there is interference with the teeth’s closure pathway, the mandible must avoid aberrant contact locations along the journey from the first contact to its final secure position of tooth. As a result, it took longer to get from the first point of contact to the intercuspation position. Meanwhile, interferences in the trajectory of the mandibular marginal movements such as excursion and protrusion can prolong the time taken for teeth to separate. Variations in tooth contacts over time can thus represent occlusal movement abnormalities. Occlusal separation time was a common metric for evaluating occlusal [12]. The T-Scan can also reveal the occlusal force distribution, the existence of aberrant force concentrating zones, and much more. The accuracy and reproducibility of the approach have been verified by several investigations, hence clinical practitioners have recognized it as valid and useful for investigating dynamic [11, 13].

In Andrews’ opinion, achieving an excellent result in static occlusion was likely to lead to the achievement of functional occlusal [14]. An acceptable/good appearance of teeth alignment does not always imply a ‘perfect’ functional occlusion, according to studies in post-orthodontic [15, 16]. It was discovered in a study using the T-Scan system that some patients who achieved orthodontic treatment with normal arch morphology had a higher risk of occlusal interference during functional movements than patients with natural normal occlusion, or without significant improvement compared to their pre-treatment [17], though the alignment had already been corrected. Much of the attention on orthodontic treatment has concentrated on achieving static appearance goals. However, less attention has been given to dynamic motion. Disorders in dynamic function have been verified related to unnecessary tooth wear, future temporomandibular joint disorders (TMDs), myofascial pain and relapse, etc. Orthodontic treatment can have a significant impact on the static occlusal contact relationship as well as on dynamic movement. Therefore, it is necessary to evaluate the completion quality of cases through quantitative indicators for both static and dynamic occlusion, as opposed to subjective judgment to determine whether the criteria of orthodontic treatment have been [18]. Meanwhile, the correlation between the items of static occlusion and dynamic occlusion remain undetermined. It was still unclear how the static category affects the dynamic function.

Herein, this study was proposed to quantify the two occlusion states in a group of patients who had completed orthodontic treatment, as well as to analyze the internal association between the static and dynamic occlusion using the selected parameters. Efforts have been made in this study to evaluate the viability of two scoring systems and emphasize the necessity of examinations for both static and dynamic occlusion for clinical practice.

## Materials and methods

### Participants

The study was approved by the Ethics Committee of the Chinese PLA General Hospital (PLAGH). Before beginning, each patient was given both oral and written information, and all subjects signed the consents. The study was performed in accordance with the ethical standards as laid down in the 1964 Declaration of Helsinki and its later amendments or comparable ethical standards.

Initial imaging and a detailed clinical examination were performed to identify subjects who met the following criteria: (1) treatment with the two-arch fixed orthodontic appliance without extractions or auxiliary appliances; (2) normal tooth alignment that meets the clinical criteria for treatment completion assessed by orthodontic experts; (3) natural permanent dentition without missing teeth except third molars; (4) no large restorations on the occlusal surfaces of posterior teeth nor dental implants; (5) normal skeletal relationship measured in sagittal, vertical and transverse dimensions.

Patients with (1) an overall ABO-OGS score over 27, (2) previous experience of orthodontic treatment, (3) a severe skeletal deformity (ANB<-6° or ANB>6°, protrusion or recession of maxillary/mandibular bone greater than 3 mm), (4) severely worn dentition, and (5) symptoms related to temporomandibular joint disorders or severe periodontal disease were excluded from this study.

A total of 112 consecutively treated patients by the same experienced orthodontic clinician at PLAGH were included in this study, consisting of 64 females (57.1%) and 48 males (42.9%). The demographic characteristics of the samples are described in Table 1. Based on the pre-treatment Angle’s classification of the malocclusion, samples were divided into 4 groups: the Angle I group, the Angle II^1^ group, the Angle II^2^ group (combined as the Angle II group), and the Angle III group.

**Table 1:** Demographic data of subjects

Characteristics	Data
Number (n, %)	112(100%)
Age (Mean ± SD years)	25.68 ± 4.70
Treatment duration (months)	20.39 ± 2.52
**Gender** (n, %)	
Female	64(57.1%)
Male	48(42.9%)
**Molar Relationship Classification** (n, %)	
Angle I	35(37.8%)
Angle II	44(39.3%)
Angle II^1^	31 (27.7%)
Angle II^2^	13 (11.6%)
Angle III	33(29.5%)

### ABO scoring

According to the standards of the objective grading system of the American Board of Orthodontics (ABO-OGS), the points of discrepancies of each tooth were scored from 0 to 2 points in each category and summed up for an overall score to represent the quality of the case. Large divergence from the norm indicated more points of item, showing its poor performance. ABO criteria were met if the patients’ overall score of 8 categories is less than 20. Cases with a score of 20–30 were considered to have the potential of passing the board. If the case receives more than 30 points, it is considered unacceptable. The cutoff score is lower if root angulation is not taken into account. In research determining how well the OGS measured the complete quality of adult patients excluding assessment of radiographs, the cutoff score was 27 [19]. Dental casts and occlusal records were taken as soon as the orthodontic appliances were removed. To replicate the virtual occlusal condition of patients, a facebow (ARCUS, Kavo, Germany) record was acquired and transferred to a full adjustment articulator (PROTAR evo7, Kavo, Germany). All dental models derived from patients were measured and scored using the ABO-OGS (Revised in 2012) and the specific ABO measuring gauge (Fig. 1). In the meanwhile, the cutoff score is 27 in this study. The score for each item was measured 3 times by the same examiner and averaged. All ABO-OGS indices were measured by the same physician who had taken the professional training courses provided by the American Board of Orthodontics and with adequate experience.

### T-Scan examination

The examination was finished on the same visit as the removal of orthodontic appliances. All subjects were taught the required mandibular motions before the examination, including centric occlusion, protrusion, and lateral excursions. Patients were instructed to practice the movements until they could be performed fluently, and the examination began after a 10-minute break. The mesiodistal width of the maxillary teeth was measured by a digital vernier caliper (Mitutoyo, Kanagawa, Japan) and imported into the T-Scan system (version 10, Tekscan, Inc., Norwood, MA, USA) for an individual dental arch dimension. Patients were instructed to sit upright while the Frankfort-Horizontal plane was parallel to the ground during the examination. To reduce measurement errors, each movement was measured three times, and a five-minute break was allowed between each type of movement to avoid muscle and joint fatigue. Patients were seated in standard position and the position of the sensor remains unchanged in each examination. Variables of the T-Scan system are described in Table 2. A T-Scan display screen presenting the distribution of occlusal force is shown in Fig. 2 (Fig. 2c). All T-Scan examinations were performed strictly under instructions and supervision of the same physician.

**Table 2:** Definition and Descriptive data of the T-Scan Variables

Variables	Definition	Mean	SD	Min	Max
OT (s)	Occlusion time at maximum intercuspal position (MICP)	0.211	0.079	0.060	0.410
DT-L (s)	Disocclusion time at left excursion movement	0.821	0.320	0.200	1.460
DT-R (s)	Disocclusion time at right excursion movement	0.784	0.326	0.290	1.760
DT-P (s)	Disocclusion time at protrusion movement	0.666	0.283	0.240	1.430
Ratio of force distribution (percentage, %)					
DOF-A (%)	Force distribution of incisors and canines at maximum intercuspation	27.45	11.88	9.54	53.02
DOF-P (%)	Force distribution of premolars and molars at maximum intercuspation	57.42	16.67	21.30	84.10
DOF-L (%)	Force distribution of Left maxillary region	47.69	8.40	33.00	73.00
DOF-R (%)	Force distribution of Right maxillary region	52.31	8.40	27.00	67.00
AOF (%)	Asymmetry index of occlusal force, AOF AOF= (Force of left side-Force of right side)/Total force×100%	-1.35	11.01	-26.97	15.2
Force (kg)	Maximum occlusal force at MICP	77.17	29.24	26.60	140.01
Area (mm^2^)	Area of occlusal contact surface at MICP	311.26	96.11	154.20	495.89

### Statistical analysis

Data analyses were performed by SPSS version 23.0 for windows (SPSS Inc, Chicago, IL, USA). The means, standard deviations (SD), and ranges of each item were calculated using standard descriptive statistics. Because ABO-OGS scores are discontinuous grading numbers, non-parametric tests such as Kruskal-Wallis were used to compare differences between groups. After the normality test, the T-Scan system results conformed to the normal distribution, so they were tested using one-way ANOVA tests, followed by a posthoc analysis using the least significant difference (LSD) test to compare the differences of the same item between different groups. The Spearman test was also applied to analyze the correlation between ABO-OGS parameters and T-Scan variables. The statistical significance level was set at p < 0.05.

### Reliability test

From the total of 112 subjects, 20 were selected at random for a re-examination of the same index by the same examiner after 2 weeks. The Wilcoxon signed-rank test was utilized to assess the reproducibility of T-Scan results, and the random error of ABO-OGS measurements and T-Scan variables was calculated using Dahlberg’s formula: $Se=\sqrt {\frac{{\sum {{d^2}} }}{{2n}}}$, where d represents the difference between the two measured values and n represents the number of subjects.

## Results

Based on the results of the reliability tests, neither the ABO-OGS score nor the T-Scan variables exhibited any systematic error (Table 3). The level of random error during the model measurements was within an acceptable range and did not have a significant impact on the accuracy of the analysis of the experimental results or the derivation of the study conclusions.

**Table 3:** Reliability test of the ABO-OGS and T-Scan results

Category	Random Error	Category	Z	P value	Random Error
ABO-OGS scores	0.281	OT	-0.951	0.128	0.021
Alignment	0.926	DT-L	-1.470	0.265	0.047
Marginal Ridges	0.727	DT-R	-1.254	0.108	0.037
Buccolingual Inclination	0.444	DT-P	-1.301	0.332	0.049
0verjet	1.192	DOF-A	-1.113	0.393	0.041
Occlusal Contact	0.944	DOF-P	-0.215	0.856	0.060
Occlusal Relationship	0.438	DOF-Left	-0.924	0.960	0.027
Interproximal Contact	0.032	DOF-Right	-0.851	0.443	0.028
		AOF	-1.070	0.261	0.014
		Force	-0.954	0.238	7.908
		Area	-0.403	0.549	8.267

**Fig. 3:**
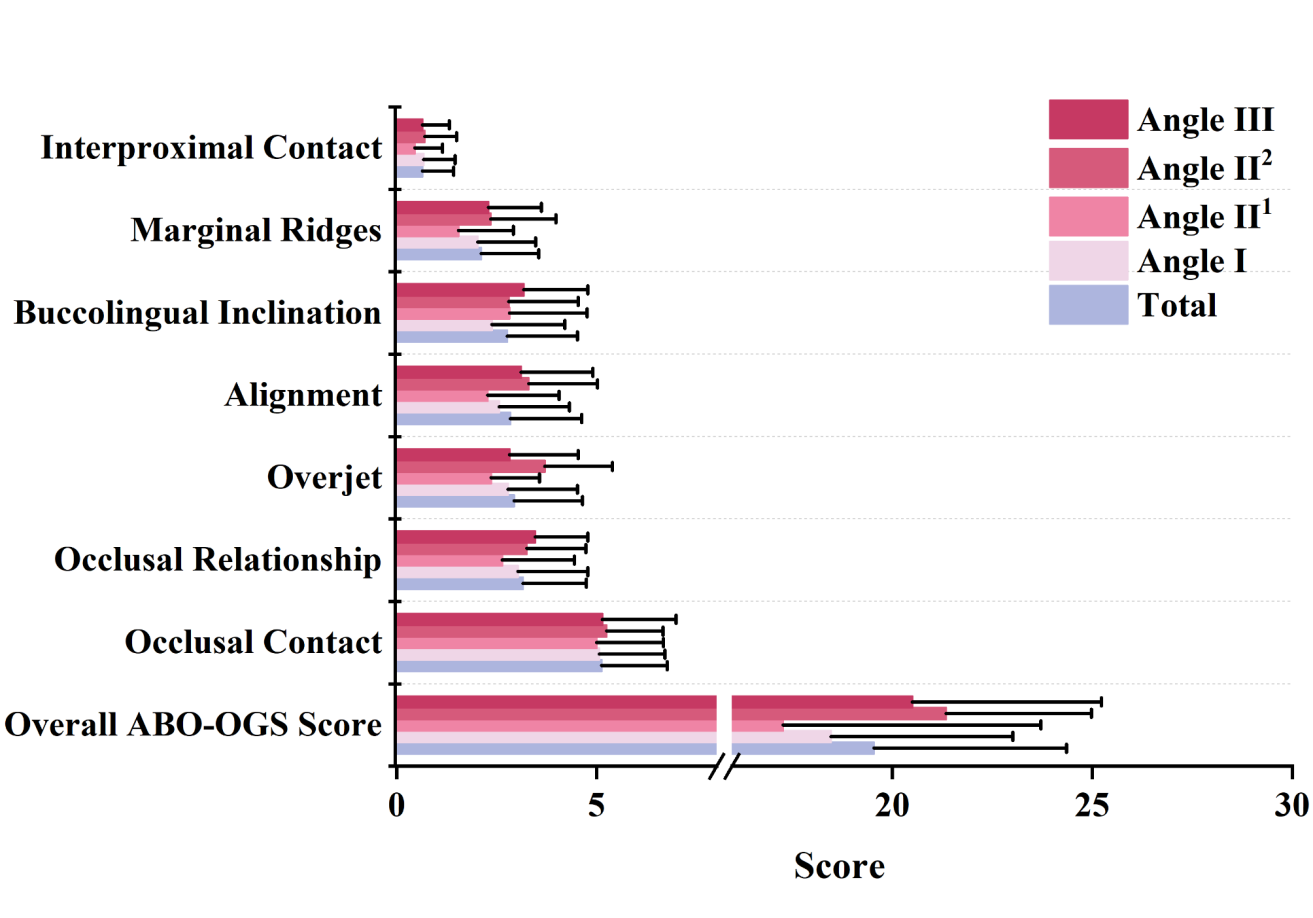
The score for each category and the overall ABO-OGS score. There was no significant difference in the score of static variables between different Angle classifications. The contributions of various categories of static morphology to the ABO-OGS score were ranked as follows: occlusal contacts > occlusal relationships > overjet > alignment > buccolingual inclination > marginal ridges > interproximal contacts

The total ABO-OGS score and other score components did not differ significantly between patient classifications (Fig. 3). Therefore, the Angle II^1^ group and Angle II^2^ group were combined as the Angle II group for the discussion of static characters. The categories of static morphology contributing most to the ABO-OGS score were ranked as follows: occlusal contacts (26.20%) > occlusal relationships (16.17%) > overjet (15.05%) > alignment (14.53%) > buccolingual inclination (14.12%) > marginal ridges (10.80%) > interproximal contacts (3.28%). Using a single sample t-test, the average ABO-OGS total score of 19.54 ± 4.82 does not differ significantly from the cut-off value of 21 (p = 0.023, 95%CI=-2.531- -0.197).

**Fig. 4:**
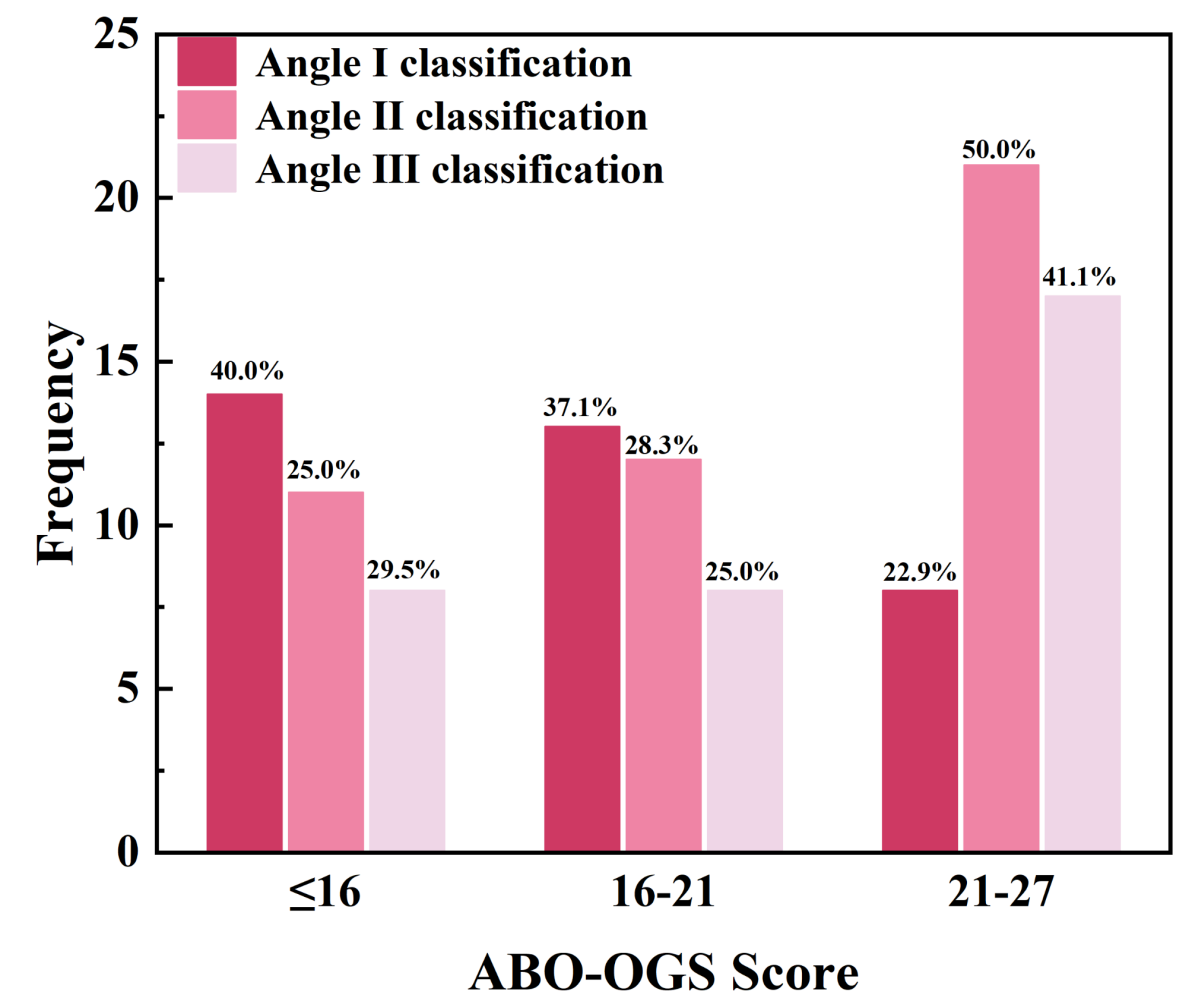
Composition of overall ABO-OGS in patients with different Angle classifications. The majority of Angle I samples (77.1%) scored below 21, while 22.9% scored between 21 and 27. In the Angle II classification, 47.7% of the participants scored between 21 and 27, 53.3% scored below 21, and 25.0% scored below 16. In the Angle III classification, half scored between 21 and 27, 50% scored less than 21, and 25% scored less than 16

The overall mean value of overall ABO-OGS score was ranked as follows: Angle I (18.47 ± 4.54) < Angle II (19.90 ± 5.11) < Angle III (20.50 ± 4.73), p > 0.05. The majority of Angle I samples (77.1%) scored below 21, while 22.9% scored between 21 and 27. In the Angle II classification, 47.7% of the participants scored between 21 and 27, 53.3% scored below 21, and 25.0% scored below 16. In the Angle III classification, half scored between 21 and 27, 50% scored less than 21, and 25% scored less than 16 (Fig. 4). In total, 59% of the sample received a score below 21.

OT of all post-orthodontic patients in this study was 0.211 ± 0.079s (Table 2). Using the single sample t-test, there was no significant difference between OT = 0.2s in normal occlusion and the overall mean in this study (p = 0.323, 95%CI=-0.011-0.032). DT-L, DT-R, and DT-P were substantially longer than the mean value of DT = 0.5s in normal occlusion (p < 0.01, 95%CI for DT-L = 0.235–0.407, 95%CI for DT-R = 0.089–0.242, 95%CI for DT-P = 0.195–0.372). DOF-R was larger than DOF-L using the pairwise t-test (p = 0.046).

**Fig. 5:**
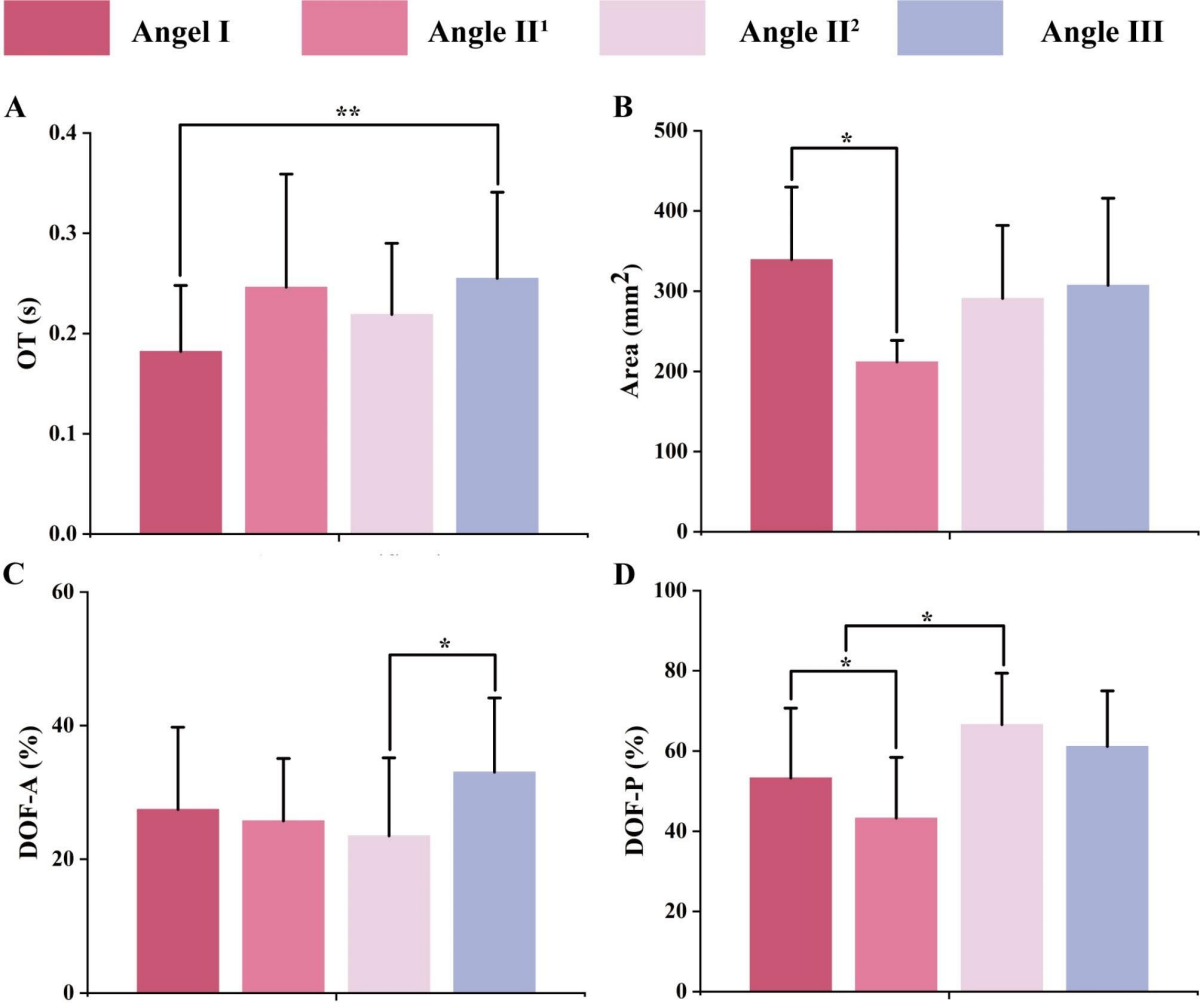
Differences in dynamic occlusion indices that differed in different Angle classifications tested by LSD. (a) The OT of Angle III patients was significantly longer than the OT of Angle I patients (p = 0.008). (b) The area of occlusal contact of Angle I patients was found to be larger than Angle II^1^ patients (p = 0.013). (c) DOF-A of the Angle III group was found significantly larger than the Angle II^2^ group (p = 0.005). (d) DOF-P of the Angle II^2^ group was larger than the Angle II^1^ group (p = 0.011) and the Angle I group (p = 0.013)

The differences between groups of T-Scan system results were determined by one-way ANOVA test and followed by the post hoc analysis by least significant difference (LSD) test (Fig. 5). OT of Angle III patients was observed significantly longer than OT of Angle I patients (p = 0.008). DOF-A of the Angle III group was found significantly larger than the Angle II^2^ group (p = 0.005). DOF-P of the Angle II^2^ group was larger than the Angle II^1^ group (p = 0.011) and the Angle I group (p = 0.013). Area of occlusal contact of Angle I was found to be larger than Angle II^1^ (p = 0.013). According to the pairwise t-test, DOF-R was significantly larger than DOF-L (p = 0.046).

**Fig. 6:**
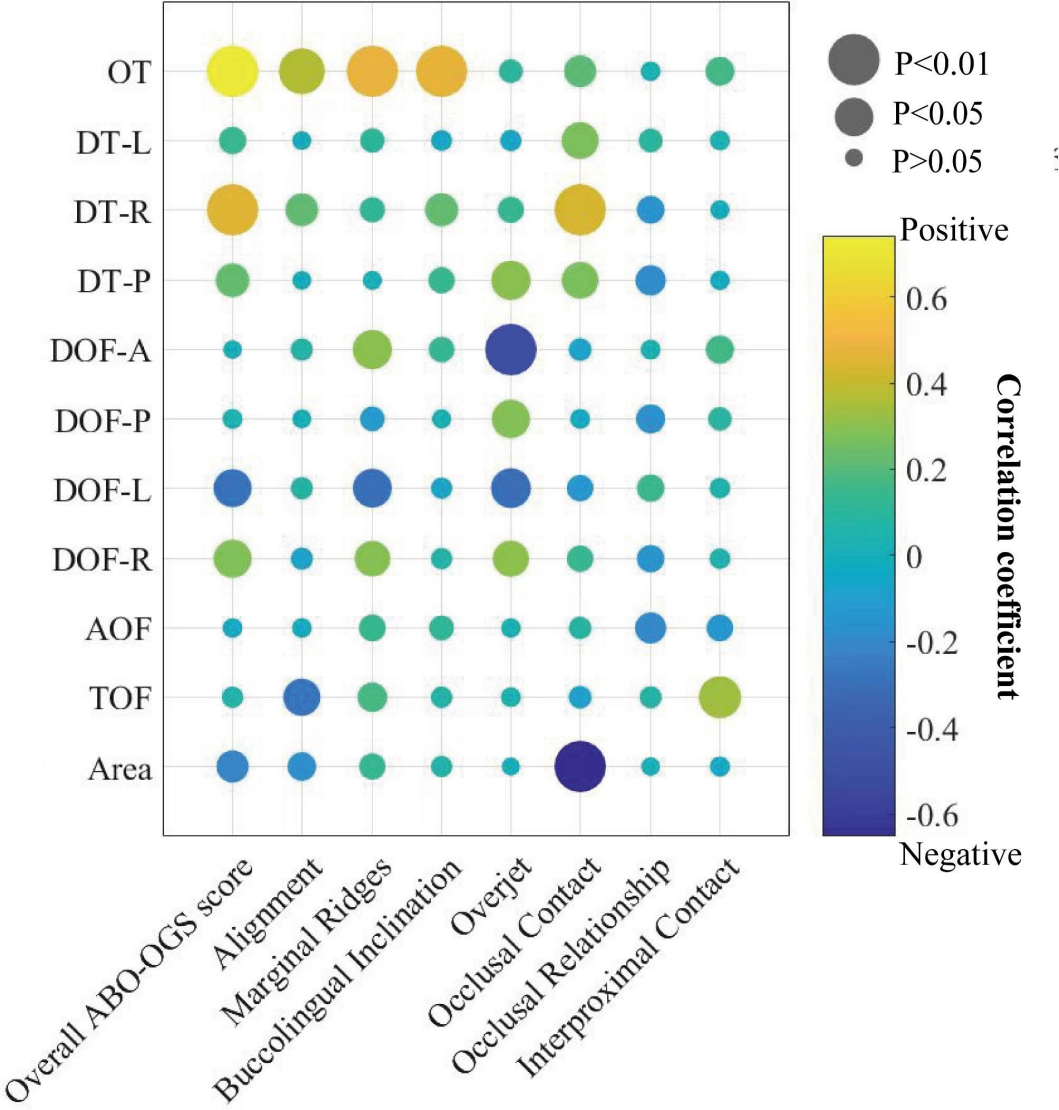
Heatmap of spearman correlation between T-Scan and ABO-OGS variables. The X-axis represents the static occlusal index, and the Y-axis represents the dynamic occlusal index. As shown above, the correlation between dynamic and static indices was presented by the colored dots. The color of the dot represents the correlation coefficient, and the value was shown by a gradient color band. The p-value is represented by the diameter of the dot. The larger the p value is, the smaller the diameter of the bubble is. The statistical significance level was set at p < 0.05. The depth of the color indicates the magnitude of the correlation value, which corresponds to the correlation color column on the right side of the heat map. According to the heat map, OT of occlusal contact time, DT of occlusal separation time, DOF-A of anterior teeth, DOF-P of posterior teeth, TOF of bite force and Area of occlusal contact were T-Scan variables with significant correlation with ABO-OGS index

Spearman correlation analysis was applied to further determine the correlation between static and dynamic occlusal variables (Fig. 6). OT, DT, DOF-A, DOF-P, force, and contact area are T-Scan variables that are highly correlated with ABO-OGS indices. There was a significant positive correlation between overall ABO-OGS score and OT (r = 0.742, p < 0.01), as well as DT-R (r = 0.456, p < 0.01). Same results were also seen between alignment and OT(r = 0.370, p < 0.01), between marginal ridges and OT (r = 0.483, p < 0.01), DOF-A (r = 0.298, p = 0.027), between buccolingual inclination and OT (r = 0.472, p < 0.01), between overjet and DT-P (r = 0.300, p = 0.026), DOF-P(p = 0.033), between occlusal contacts and DT-L (r = 0.271,p = 0.045), DT-R (r = 0.442, p < 0.01), DT-P (r = 0.270, p = 0.047), and between interproximal contacts and force (r = 0.334, p = 0.013). Nevertheless, a negative correlation was found between alignment and force (r=-0.274, p = 0.043), between overjet and DOF-A (r = 0.499, p < 0.01), and between occlusal contacts and contact area (r=-0.652, p < 0.01).

## Discussion

Through the results of the current study, it was found that passing the clinical examination and the evaluation of static casts does not equate to dynamic function perfection. Prolonged DT was detected in the post-orthodontic patients, indicating there is drag and friction present in excursions on many posterior teeth, as the DT measures the sum of all molar and premolar, working and balancing excursive contact durations. Differences in the occlusal contact area and force distribution in MIP were identified in patients of different Angle classifications. Internal correlations were found between dynamic motions and static indices. Occlusion time, disocclusion time, and distribution of force in dynamic motions were considerably influenced by static performance, particularly occlusal contacts, buccolingual inclination, and alignment of teeth.

The samples included were from patients with different horizontal and vertical bone facial type and is not a completely homogeneous sample; therefore, the conclusions obtained from this study are representative for post-orthodontic patients with different classifications. In this study, all subjects were trained beforehand to guarantee that they had learned the necessary mandibular motions before T-Scan data was collected. To ensure the precision and the dependability of the research findings, the systematic error and random error of outcomes were measured. Both ABO-OGS scores and T-Scan items demonstrated adequate repeatability.

The ABO-OGS is a valid and reliable index that is applicable worldwide. ABO has established detailed guidelines and a standardized tool or scoring instances under various circumstances. For individuals with varied molar and incisor relationships, for instance, scores of occlusal relationships are determined based on a distinct reference position, given the fact that they all show an acceptable relationship between the positions of the upper and lower arches. As an evaluation criterion, ABO-OGS represents the static morphology of cases recognized by physicians and their probability of meeting the requirements. This grading system is more straightforward and less susceptible to human involvement, which could be influenced by artificial weighting or subjective prejudice of examiners. The evaluation of the ABO-OGS is congruent with the clinician’s criteria in determining whether orthodontic treatment is successful or not.

All of the samples included in this study were assessed by orthodontic doctors to have achieved normal or optimal tooth morphology and function and met the criteria for completion of orthodontic treatment. The traditional view holds that Angle Class I malocclusions appear to have an advantage in achieving passing ABO-OGS [20]. In this study, the overall mean value was ranked as follows: Angle I < Angle II < Angle III, whereas no statistically significant differences were identified between pretreatment Angle classifications. It was inferred that patients with different classifications have the same probability of passing ABO-OGS evaluation after orthodontic treatment. The objectivity of ABO-OGS as a universal quality evaluation system was further verified. In a regional study validating the ABO-OGS system for assessing Chinese patients, a satisfactory treatment outcome was defined as 16 points or less, scores of 16–21 were less satisfactory but still acceptable and cases scoring more than 21 were considered as [21]. Comparing the overall case qualification to regional standard cases in this study, this cutoff value of 21 was met.

The composition of the ABO-OGS scores may include occlusal contacts, overjet, occlusal relationships, and alignment account for the greatest proportion of points. As predicted by previous studies, occlusal contacts appeared to be the category where ABO-OGS certification cases received the highest [20]. When analyzing the static morphology and inter-arch relationship of post-orthodontic cases, the performance of the occlusal contacts and occlusal relationship is of crucial importance. Consistent with the opinions of Chaison et al., in their study of adult orthodontic patients evaluated by ABO-OGS, the primary factor that influences the quality of a completed case as determined by professionals is whether adequate intercuspation is [19]. Conversely, these items received the highest scores among the seven items, suggesting that clinical practitioners might lack control over these items. Though the settling during the retention period might increase occlusal contact, this procedure is fraught with uncertainty and companies by tooth [22, 23]. Interproximal contacts had the lowest score, indicating that the majority of orthodontic cases performed well in closing the excess space, which was relatively the easiest part of orthodontic treatment to control.

Through decades of research, the T-Scan system has been regarded as a trustworthy approach for measuring mandibular [13]. OT values less than 0.2s are considered normal, while DT values less than 0.5s are considered normal. Time spent more than the normal value is considered an indicator of early contacts in the central occlusion or occlusal interference during lateral excursions and protrusions. In this study the OT of the Angle I group was smaller than the Angle III group, revealing that Angle I patients were more likely to obtain a smooth central occlusion without interference through orthodontic treatment than Angle III patients. Consistent with the results of [13], the increase of DT in post-orthodontic patients in this study was observed compared to the overall mean value in normal occlusion from recognized studies.

All cases were valued before tearing down the appliances and succeeded in the chair-side functional examination using articulating paper or foils—the most commonly-used materials in clinical practice. There still exists minor interference in the trajectories of mandibular movements which failed to be detected. The sensitivity and reliability of traditional methods rely on the thickness and elasticity of the occlusion-checking materials; nevertheless, the result can be easily impacted by the oral environment and the interpretation of clinician. Sutter observed that the subjective interpretation of occlusal paper impression resulted by dentists had a correct rate of 13.13%, which was even lower than the randomly computed correct rate of 16.7% [24]. This omitted imperfection of occlusion may not interfere with the overall clinical assessment or cast measurements. However, those minor interference might potentially lead to unnecessary wear of teeth, tooth sensitivity, TMDs, myofascial pain, pathological tooth mobility, relapse, [25, 26]. The prolonging of OT and DT had been confirmed as an indicator of [27, 28]. According to some research, post-orthodontic patients harboring “normal occlusion” had a higher prevalence of TMDs than those with natural normal occlusion or untreated minor [27]. This was attributed to the high probability of the existence of occlusal interference in the posterior teeth area in post-orthodontic occlusions. Nevertheless, occlusal interference in the posterior region is difficult to detect due to visual field restriction or moisture on the occlusal surface. With the aid of the T-Scan system whose sensitivity is 0.003s, obstructions in the movement trajectory can be easily detected from the front and rear. The T-Scan technology is advantageous throughout both the evaluation and therapy [13]. T-Scan-assisted occlusion adjustment that reduces DT may significantly relieve the myofascial syndrome including muscle pain and [29–31]. Before deciding to remove the orthodontic device, it is highly recommended that a thorough analysis of the functional mandibular movements is performed using the T-Scan system.

Angel classifications might have an impact on occlusal contact after orthodontic treatment. Al-Rayes et al. found that the occlusal contact area of Angle II^2^ patients was smaller than normal subjects, with no significant difference, while Angle I patients had the highest number of occlusal contacts compared to other malocclusion [32]. This study depicted that the occlusal contact area of Angle I patients was significantly greater than Angle II^2^ patients. It is logical to expect that the relative recession of the mandibular molar position is the reduction in the contacting area between the maxillary and mandibular arches.

The variation of force and contact area is large among individuals. Consequently, comparing the absolute value between individuals might be misleading. Instead, the concentration should be on indicators with fewer individual variations, such as the distribution of occlusal force (DOF), the asymmetry index of occlusal force (AOF), masticatory efficiency, etc. Through the DOF indicated by T-Scan, it was determined that whether the occlusal force distribution is in equilibrium and where excessive force concentrations exist. In this study, DOF-A of the Angle III group was larger than the Angle II^2^ group, and DOF-P of the Angle II^2^ group was larger than Angle III and Angle I groups. The occlusal force was mainly concentrated in the posterior region in centric occlusion ranging from 21.3 to 84.1%. Orthodontic treatment might increase the frictional contacts in the posterior [27]. According to the study of Qadeer, the force distribution in the posterior region in post-orthodontic patients was 89.42% and was significantly larger than 77.57% in non-orthodontic [33]. The force distribution of the right maxillary region was statistically larger than on the left side. According to the study supported by Yamada et al., the dominant occlusal force side in normal occlusion people using the T-Scan system was the right side, while the asymmetry index was 9.2% [34], which was compatible with the findings. Makino et al. concluded that orthodontic treatment could improve the distribution of occlusal forces, making them more balanced and increasing the occlusal contact area, resulting in a better alignment of teeth with neuro-muscle function more [35].

In this study, the occlusal contact area was found to be negatively related to the score of occlusal contacts. As the adequacy of the contact between premolars and molars decreases, the area of contact decreases accordingly. The value of occlusal force was not directly related to the Angle classifications in normal vertical skeletal patterns but was determined by different individuals. Correlations were found between force and alignment, suggesting that the coordination of teeth alignment may influence the occlusal force applied.

Appropriate cusp inclination, sufficient cusp height, and an adequate occlusal contact area together guarantee reasonable masticatory function. The sequential occlusal separation of the anterior and posterior teeth during lateral movement is a prerequisite for a stable and uninterrupted occlusal movement. The smoothness of the dynamic mandibular trajectory is highly associated with the static morphology indices and is able to change accordingly. Abnormalities in the static occlusion can be reflected in the smoothness of the dynamic movements. Sierpinska et al. found that occlusal surface morphologies of premolars may have a direct impact on occlusion [36]. In this study, a positive correlation was found between OT and ABO-OGS score, alignment, marginal ridges, and buccolingual inclination. The results showed that as the scores of ABO-OGS, alignment, marginal ridges, and buccolingual inclination increased, the evaluation of static occlusion became worse and the occlusion time increased accordingly, further indicating the smoothness of dynamic occlusion was interfered with by these characters. It was suggested that the time required from the first tooth contact to the maximum intercuspal occlusion in post-orthodontic patients was positively correlated with the neatness of static tooth alignment, the consistency of marginal ridge height, and the correct buccolingual inclination of the maxillary and mandibular posterior teeth. There was a positive correlation between the disocclusion time of teeth during mandibular protrusions and excursions and occlusal contacts. As the score of occlusal contacts increased, the adequacy of occlusal contact of posterior teeth became worse and the separation time of the non-working side teeth in lateral mandibular excursion movements or the posterior teeth as the mandible move forward became longer. This represented that when there was a deviation in the working-side/non-working-side tooth cusp-fossa relationship, resulting in offset or obstruction in the path when the mandibular teeth slide along the cusp bevel, the time required for the non-working-side teeth to detach from contact was prolonged. Orthodontic treatment was related to the increase of frictional contacts in the non-working side and posterior region. Meanwhile, DT-P and DOF-P had a positive correlation and DOF-A had a negative relation with overjet. The increase in the score for overjet indicated the distance between the upper and lower canines and incisors increased. Therefore, as the score of overjet increased, the force distribution of anterior teeth decreased and the time taken for the mandibular protrusion along the guidance of the lingual surface of maxillary canines and incisors prolonged. The increase in the distribution of the posterior region might be due to the decrease in the anterior supporting.

The higher score of variables on the ABO-OGS may be indicative of poor occlusal contact performance. The aberrant contact observed in static models may result in a longer path for the mandible during protrusions, interference during lateral excursions, and early contact during intercuspal occlusion. The T-Scan system detects tiny aberrations in dynamic motions by identifying longer duration for dynamic motions, anomalous force distributions, and relative contact areas. This study confirmed that a “successful” static occlusion does not equate to dynamic function perfection. Though the overall performance of the case may still pass the evaluation both on the dental chair and on the cast after treatment, prospective repercussions must be addressed. Significant correlations were found between the static categories and dynamic variables. Occlusal interference was detected by the T-Scan system in cases whose scores of occlusal contacts, overjet, and alignment were relatively high. It was confirmed that the greater the quality of the orthodontic finished occlusion, the better the occlusal status at the post-retention [37]. Therefore, clinicians should pay extra attention to these items and whether the dynamic occlusion has been compromised when these categories score abnormally.

The morphology and function of the oral system consist of multiple components that are mutually restricted and coordinated. Through the unfavorable occlusal contacts reported in this study, guidelines and standards for appropriate dynamic occlusion should be addressed. The current study did not examine the functions of TMJ. Acknowledging this limitation, we intend to investigate optimal occlusion standards for post-orthodontic patients, which may accomplish not only aesthetic appearance but also interference-free function. Smooth occlusion refers to the static smoothness of teeth alignment, ridges, relationships, curves and the fluidity of dynamic movements. More reasonable variables should be added to the evaluation of post-orthodontic occlusion, including the occlusion and separation time of teeth, teeth contact pattern during movement, the trajectory of the mandible, the discrepancies between intercuspal position, and central relation position. Standardization of orthodontic assessment criteria facilitates further development of the discipline. Moreover, the development of digital technology might bring a digital flow for orthodontic treatment, including CBCT, 3D casts, virtual articulators, computerized axiography, [37, 38]. The application of multi-modal fusion in the evaluation of oral morphology and function is also of promising potential in the diagnosis and treatment of the orthodontic field.

## Conclusions

This study indeed confirmed the validity and accuracy of the ABO-OGS and the T-Scan system as two occlusal assessment modalities. The two approaches are considered available to objectively and quantitatively evaluate the quality of post-orthodontic patients in both static and kinetic dimensions. However, passing the clinical examination and the evaluation of static casts does not equate to dynamic function perfection. Internal correlations were found between dynamic motions and static indices. Occlusion time, disocclusion time, and distribution of force in dynamic motions were considerably influenced by static performance, particularly occlusal contacts, buccolingual inclination, and alignment of teeth. Therefore, both static and dynamic occlusion should be extensively evaluated before the removal of the orthodontic appliances. It is recommended that guidelines and standards for appropriate dynamic occlusion evaluation should be explored in the future.

## Data Availability

The data set supporting the conclusions of this article are included within the article. Further data sets are available from the corresponding author upon reasonable request.
